# Association of Body Mass Index in Midlife With Morbidity Burden in Older Adulthood and Longevity

**DOI:** 10.1001/jamanetworkopen.2022.2318

**Published:** 2022-03-15

**Authors:** Sadiya S. Khan, Amy E. Krefman, Lihui Zhao, Lei Liu, Anna Chorniy, Martha L. Daviglus, Cuiping Schiman, Kiang Liu, Tina Shih, Daniel Garside, Thanh-Huyen T. Vu, Donald M. Lloyd-Jones, Norrina B. Allen

**Affiliations:** 1 Division of Cardiology, Department of Medicine, Northwestern University Feinberg School of Medicine, Chicago, Illinois; 2 Department of Preventive Medicine, Northwestern University Feinberg School of Medicine, Chicago, Illinois; 3 Division of Biostatistics, Washington University in St Louis, St Louis, Missouri; 4 Department of Medical Social Sciences, Northwestern University Feinberg School of Medicine, Chicago, Illinois; 5 Institute for Minority Health Research, College of Medicine, University of Illinois at Chicago, Chicago; 6 Department of Economics, Georgia Southern University, Statesboro

## Abstract

**Importance:**

Abundant evidence links obesity with adverse health consequences. However, controversies persist regarding whether overweight status compared with normal body mass index (BMI; calculated as weight in kilograms divided by height in meters squared) is associated with longer survival and whether this occurs at the expense of greater long-term morbidity and health care expenditures.

**Objective:**

To examine the association of BMI in midlife with morbidity burden, longevity, and health care expenditures in adults 65 years and older.

**Design, Setting, and Participants:**

Prospective cohort study at the Chicago Heart Association Detection Project in Industry, with baseline in-person examination between November 1967 and January 1973 linked with Medicare follow-up between January 1985 and December 2015. Participants included 29 621 adults who were at least age 65 years in follow-up and enrolled in Medicare. Data were analyzed from January 2020 to December 2021.

**Exposures:**

Standard BMI categories.

**Main Outcomes and Measures:**

(1) Morbidity burden at 65 years and older assessed with the Gagne combined comorbidity score (ranging from −2 to 26, with higher score associated with higher mortality), which is a well-validated index based on *International Classification of Diseases, Ninth Revision* codes for use in administrative data sets; (2) longevity (age at death); and (3) health care costs based on Medicare linkage in older adulthood (aged ≥65 years).

**Results:**

Among 29 621 participants, mean (SD) age was 40 (12) years, 57.1% were men, and 9.1% were Black; 46.0% had normal BMI, 39.6% were overweight, and 11.9% had classes I and II obesity at baseline. Higher cumulative morbidity burden in older adulthood was observed among those who were overweight (7.22 morbidity-years) and those with classes I and II obesity (9.80) compared with those with a normal BMI (6.10) in midlife (*P* < .001). Mean age at death was similar between those who were overweight (82.1 years [95% CI, 81.9-82.2 years]) and those who had normal BMI (82.3 years [95% CI, 82.1-82.5 years]) but shorter in those who with classes I and II obesity (80.8 years [95% CI, 80.5-81.1 years]). The proportion (SE) of life-years lived in older adulthood with Gagne score of at least 1 was 0.38% (0.00%) in those with a normal BMI, 0.41% (0.00%) in those with overweight, and 0.43% (0.01%) in those with classes I and II obesity. Cumulative median per-person health care costs in older adulthood were significantly higher among overweight participants ($12 390 [95% CI, $10 427 to $14 354]) and those with classes I and II obesity ($23 396 [95% CI, $18 474 to $28 319]) participants compared with those with a normal BMI (*P* < .001).

**Conclusions and Relevance:**

In this cohort study, overweight in midlife, compared with normal BMI, was associated with higher cumulative burden of morbidity and greater proportion of life lived with morbidity in the context of similar longevity. These findings translated to higher total health care expenditures in older adulthood for those who were overweight in midlife.

## Key Points

**Question:**

What is the association between body mass index (BMI; calculated as weight in kilograms divided by height in meters squared) in midlife and morbidity burden in older adulthood (≥65 years) and longevity?

**Findings:**

In this cohort study of 29 621 adults, being overweight and having classes I and II obesity compared with having a normal BMI at a mean age of 40 years were associated with a statistically significantly higher cumulative morbidity score and health care costs across older adulthood. Age at death was similar in the overweight and statistically significantly younger in the classes I and II obesity group compared with the normal BMI group.

**Meaning:**

This study’s findings suggest that overweight status beginning in midlife is associated with long-term adverse health and economic consequences in the context of similar longevity.

## Teaser

This cohort study uses Medicare claims data to investigate the association between body mass index midlife and morbidity burden, longevity, and health care costs in adults 65 years or older.

## Introduction

Life expectancy in the United States declined in 2014 for the first time in more than 2 decades.^1^ The observed decline was first predicted nearly 10 years earlier in a controversial publication^2^ that cited the growing prevalence of obesity (body mass index [BMI, calculated as weight in kilograms divided by height in meters squared] >30.0) in the population as one of the greatest threats to overall health and longevity. The decline in life expectancy has been attributed in part to increasing mortality rates in the leading cause of death, cardiovascular disease (CVD).^3,4^ Despite abundant evidence that obesity is associated with higher all-cause^5,6^ and cardiovascular^7,8^ mortality, population-level prevention efforts have not been effective, and the prevalence of obesity continues to increase, with national projections estimating that nearly 1 in 2 US adults will be obese by 2030.^9^

Obesity is additionally a well-established risk factor for morbidity, including CVD,^7,8^ certain cancers,^10,11^ and other health conditions.^12^ As a result, obesity is associated with substantial health care costs, which annually exceed $140 billion.^13^ However, controversy exists regarding the relative risk of morbidity and mortality in individuals who are in the overweight status (BMI, 25.0-29.9) compared with those with a normal BMI (18.5-24.9). A systematic review and meta-analysis^14^ published in 2013 found that, relative to normal BMI, overweight was associated with lower mortality based on 97 studies including 2.88 million individuals. While abundant evidence exists for the adverse outcomes associated with obesity, the potential of a survival advantage for overweight status, if one exists, may come at the cost of a greater proportion of life lived with morbidity and, subsequently, higher health care expenditures across the life course. However, most available studies assessed BMI in older adulthood, many had less than 10 years of mean follow-up, and few assessed morbidity, mortality, and health care costs simultaneously. In light of these findings, patients in the overweight BMI category often ask whether weight loss is necessary, but direct evidence linking BMI, particularly in the overweight range, with adverse health and economic consequences over the long term and distinct from mortality is limited.

To address these unanswered questions, we examined the association of BMI in midlife with morbidity burden, longevity, and health care expenditures in older adulthood. We hypothesized that, compared with having a normal BMI, being overweight and obese in midlife would be associated with a higher exposure to morbidity, a greater proportion of life lived with morbidity (in the context of similar or shorter longevity), and greater health care costs.

## Methods

This prospective cohort study was approved by the institutional review board at Northwestern University, Chicago, Illinois. All participants signed written informed consent prior to participation. This study followed the Strengthening the Reporting of Observational Studies in Epidemiology (STROBE) reporting guideline for cohort studies.

### Study Sample

The study sample was derived from the Chicago Heart Association Detection Project in Industry (CHA) cohort, which recruited 39 665 men and women 18 years and older between 1967 and 1973. Participants have been followed up for more than 40 years since the baseline in-person examination through a variety of methods, including personal contact, National Death Index data, and administrative claims data based on linkage with Medicare files from the US Centers for Medicare and Medicaid Services (CMS) from January 1985 through December 2015. In this study, we included participants from CHA who were enrolled in the Medicare fee-for-service program and excluded individuals with missing data on covariates (risk factors or educational level) or date of birth. The study sample for this analysis included 29 621 individuals with available data on covariates who were alive and enrolled in Medicare after age 65 years (eFigure 1 in the Supplement). Of note, a similar BMI distribution was observed for those included and excluded (eTable 1 in the Supplement). The study protocol has been published.^15^ Briefly, trained personnel administered questionnaires for demographic characteristics (including self-identified race and ethnicity) and medical history. In CHA, participants could choose from the following race and ethnicity categories: Asian, Black, Hispanic, White, and other. Participants underwent in-person measurement of height, weight, heart rate, and blood pressure. Blood was collected for measurement of total cholesterol.

### Overall and Cardiovascular Morbidity Burden

Medicare fee-for-service claims data from the CMS were used from January 1985 (the first year that Medicare data were made available for public use) through December 2015. Linkage with CMS data was performed by cross-referencing Social Security number, sex, name, and date of birth for each participant.

For the primary outcome, we quantified all-cause morbidity in each year of follow-up using *International Classification of Diseases, Ninth Revision* codes to calculate the Gagne combined comorbidity score, a clinical index derived from Medicare data.^16^ This tool has been well validated in numerous administrative claims data sets as a measure of overall morbidity burden and includes comorbidities combined from the Charlson Comorbidity Index and the Elixhauser measures with a score ranging from −2 to 26 (derived from data in eTable 2 in the Supplement), with a higher score associated with higher mortality.^17,18^ For context, in the original derivation cohort, a Gagne score of 1 was associated with a 1-year mortality rate of 5.1% (95% CI, 4.9%-5.4%). For secondary outcomes, we also quantified all-cause morbidity with the Charlson Comorbidity Index as well to incorporate less severe morbidities. We also separately calculated a cumulative CVD morbidity score, which was a sum score of any CVD conditions, comprising atherosclerotic cardiovascular disease (coronary heart disease, myocardial infarction, peripheral vascular disease, and cerebrovascular disease) as well as heart failure. We focused on CVD because it is the leading cause of death in older adults (≥65 years) and because of the well-established association between BMI and morbidity related to CVD. A higher Gagne, Charlson, or CVD morbidity score reflected greater burden of morbidity. Each morbidity score was calculated per participant for every year of Medicare data until death or end of follow-up. If a participant did not have any Medicare claims during a specific year (hospitalization or outpatient visit claims), a score of 0 was assigned for that year, which may underestimate burden. However, lack of health care contact for a full year is unlikely in a participant with significant morbidity; therefore, the potential for misclassification from this decision is likely minimal.

### Health Care Costs

Health care expenditures were calculated for each individual Medicare beneficiary, including total cumulative cost in follow-up and mean annual cost (total cumulative cost divided by the years of Medicare follow-up) based on cost data from the CMS. Because Medicare is considered the primary payer for all enrollees, this includes claims paid by Medicare as well as other insurers. Therefore, claims data represent a nearly complete documentation of charges, including hospital, home health agency, hospice, skilled nursing facility, and physician expenses from inpatient and outpatient clinic visits. To account for inflation, costs were adjusted to 2016 dollars using the consumer price index for medical services published by the US Bureau of Labor Statistics. We used Medicare payment amount to approximate cost because charges can vary by region and facility.

### Statistical Analysis

We examined demographic characteristics by BMI categories at baseline. Next, we restricted the models to the participants for whom we had Medicare follow-up starting at age 65 years (n = 23 342) and who had morbidity scores of 0 at age 65 years (22 058 participants for Gagne, 22 382 for Charlson, and 22 763 for CVD morbidity). We used a penalized spline model to obtain the nonparametric estimate of the mean Gagne, Charlson, and CVD morbidity scores for each BMI category for every year of follow-up after age 65 years. A random intercept was included in the model to account for within-individual correlation. We also calculated the cumulative area under the curve for the mean Gagne, Charlson, and CVD morbidity scores during follow-up and examined differences by BMI categories. Sensitivity analyses were performed on the overall sample not restricted to a morbidity score of 0 at entry into Medicare at age 65 years for Gagne and CVD morbidity analysis.

Next, we examined the mean overall survival time (total longevity) and years lived with a Gagne, Charlson, or CVD morbidity score of 0, 1, 2, 3, or higher. Specifically, we applied the Irwin restricted mean survival time^19^ to calculate the number of years lived with a Gagne, Charlson, or CVD morbidity score of 0 or at least 1. In addition, we calculated the mean age with a Gagne score of at least 1, Charlson score of at least 1, mean age at incident CVD morbidity, and mean age at death by BMI categories. We calculated the proportion of life after age 65 years lived with a Gagne score of at least 1 (for overall morbidity) or after incident CVD morbidity across BMI categories

Third, we analyzed differences in health care costs across BMI categories compared with normal BMI as the reference group. Because medical costs are right-skewed and heteroscedastic, we used quantile regression to describe the distribution and identify the risk factors of medical costs at the 25th, 50th, 75th, and 90th percentiles.^20^ Specifically, we examined differences in cumulative and mean annual costs at different quantiles stratified by BMI. Sensitivity analyses were performed for health care costs after restriction to the sample with nonzero costs.

All models were adjusted for age, sex (when not stratified), race and ethnicity, educational level, smoking, hypertension, hyperlipidemia, diabetes measured at baseline, and whether the individual died during follow-up. Sensitivity analyses were additionally stratified by sex. All statistical analyses were performed with SAS statistical software, version 9.4 (SAS Institute Inc), and R version 3.5.2 (the R Foundation). A 2-sided *P* < .05 was considered statistically significant.

## Results

### Baseline Characteristics

A total of 29 621 participants were included with a mean (SD) age of 40 (12) years, of whom 57.1% were men, 42.9% were women, and 9.1% were Black; 46.0% had normal BMI, 39.6% were overweight, and 11.9% had classes I and II obesity at baseline. Less than 5% of the total sample consisted of Asian, Hispanic, and other races and ethnicities. Participants in the overweight group were more likely to be older (42 vs 38 years) and less likely to have more than a high school education (54.8% vs 56.3%) (Table 1). Among individuals in the overweight compared with the normal BMI category, there was a higher prevalence of diabetes (1.9% vs 1.6%), hypertension (53.6% vs 33.0%), and hyperlipidemia (18.7% vs 12.9%) but lower rates of smoking (37.4% vs 43.0%).

**Table 1.** Baseline Demographic Characteristics and Risk Factors by BMI Category in Midlife Among the 29 621 Study Participants^a^

	BMI category, No. (%)					*P* value
	Underweight (BMI, <18.5) (n = 600)	Normal (BMI, 18.5-24.9) (n = 13 638)	Overweight (BMI, 25.0-29.9) (n = 11 743)	Classes I and II obesity (BMI, 30.0-39.9) (n = 3515)	Class III obesity (BMI, ≥40.0) (n = 125)	
**Demographic characteristics**						
Age at baseline, mean (SD), y	31 (12)	38 (13)	42 (12)	43 (12)	41 (11)	<.001
Female	536 (89.3)	7893 (57.9)	3071 (26.2)	1125 (32.0)	75 (60.0)	<.001
Male	64 (10.7)	5745 (42.1)	8672 (73.8)	2390 (68.0)	50 (40.0)	
Race and ethnicity						
Black	119 (19.8)	1398 (10.3)	836 (7.1)	325 (9.2)	19 (15.2)	<.001
Educational level						
≤High school	336 (56.0)	7679 (56.3)	6439 (54.8)	2272 (64.6)	84 (67.2)	<.001
Some college	132 (22.0)	2583 (18.9)	2105 (17.9)	586 (16.7)	29 (23.2)	
College graduate	132 (22.0)	3376 (24.8)	3199 (27.2)	657 (18.7)	12 (9.6)	
**Risk factors**						
Diabetes	10 (1.7)	219 (1.6)	219 (1.9)	103 (2.9)	4 (3.2)	<.001
Current smoking	291 (48.5)	5867 (43.0)	4389 (37.4)	1213 (34.5)	45 (36.0)	<.001
Systolic blood pressure, mean (SD), mm Hg	121.2 (15.1)	129.3 (16.4)	137.3 (17.5)	144.9 (19.2)	160.5 (23.5)	<.001
Hypertension	90 (15.0)	4496 (33.0)	6292 (53.6)	2475 (70.4)	117 (93.6)	<.001
Total cholesterol,mean (SD), mg/dL	178.9 (35.6)	195.1 (39.1)	206.8 (38.0)	210.9 (39.4)	203.2 (40.9)	<.001
Dyslipidemia	34 (5.7)	1763 (12.9)	2192 (18.7)	791 (22.5)	23 (18.4)	<.001

Abbreviation: BMI, body mass index (calculated as weight in kilograms divided by height in meters squared).

SI conversion factor: To convert total cholesterol to millimoles per liter, multiply by 0.0259.

### All-Cause Morbidity in Older Adulthood by BMI in Midlife

**Figure 1.**
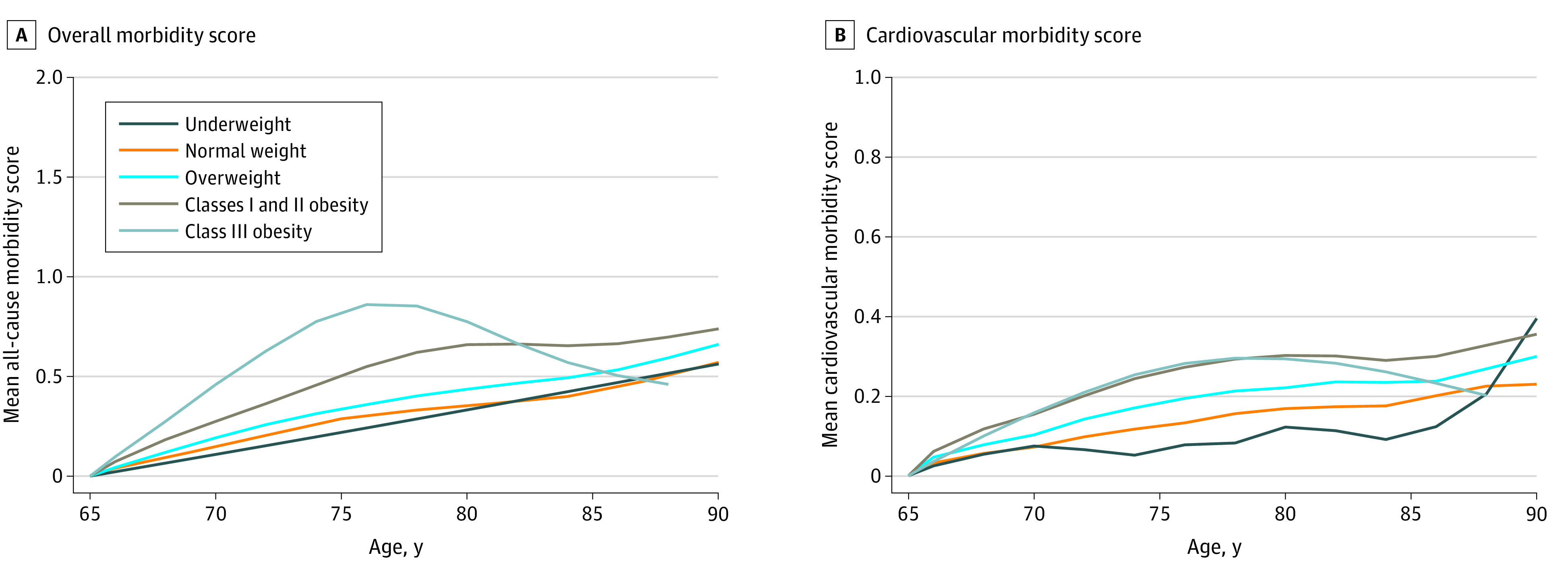
Morbidity Scores in Older Age by Body Mass Index (BMI) Category in Midlife Among those with a respective overall (A) or cardiovascular (B) morbidity score of 0 at age 65 years, annual morbidity score per participant is plotted. For all-cause morbidity, the score represents the Gagne combined comorbidity score (n = 22 058). For cardiovascular morbidity, the score represents a score of cardiovascular conditions (N = 22 763). Each line represents the midlife BMI category (calculated as weight in kilograms divided by height in meters squared), including underweight, normal BMI, overweight, classes I and II obesity, and class III obesity. Morbidity scores were adjusted for age, sex, race and ethnicity, educational level, smoking, hypertension, hyperlipidemia, diabetes, and death during follow-up.

Of those with available Medicare data, a subset had data beginning at age 65 years (n = 23 342), among whom similar demographic characteristics were observed (eTable 3 in the Supplement). Of this subset, 94.5% (n = 22 058) had a Gagne morbidity score of 0 at age 65 years. The Gagne morbidity score increased linearly with age in all BMI categories, except for participants in the class III obesity category (sometimes called morbid obesity) (Figure 1). Cumulative morbidity burden based on the Gagne score assessed per year was significantly higher, with a dose-response association in each excess BMI category of being overweight (7.22 morbidity-years), having classes I and II obesity (9.80 morbidity-years), and having class III obesity (10.32 morbidity-years) compared with the normal BMI category (6.10 morbidity-years; adjusted *P* < .001 for all comparisons; eTable 5 in the Supplement). Patterns of morbidity burden across older adulthood were similar in sensitivity analyses when the Charlson morbidity score was applied (eFigure 2 and eTable 5 in the Supplement). In sensitivity analyses, qualitatively similar findings were observed when the analysis included all participants with a Gagne score of at least 1 (eFigure 3 in the Supplement) and when participants were stratified by sex (eFigures 4 and 5 in the Supplement).

### Cardiovascular Morbidity in Older Adulthood by BMI in Midlife

In the CVD-specific analyses summing all CVD conditions per year across older adulthood, cumulative CVD morbidity burden was significantly higher, with a dose-response association in each excess BMI category of being overweight (3.84 morbidity-years), having classes I and II obesity (5.02 morbidity-years), and having class III obesity (4.15 morbidity-years) when compared with having a normal BMI (2.88; adjusted *P* < .001 for all comparisons; Figure 1; eTable 5 in the Supplement). Consistent patterns were observed for CVD when all participants were analyzed, including those with prevalent CVD at age 65 years (eFigure 3 in the Supplement) and when participants were stratified by sex (eFigures 4 and 5 in the Supplement).

### Longevity and Years Lived With Varying Morbidity Scores in Older Adulthood by BMI in Midlife

**Figure 2.**
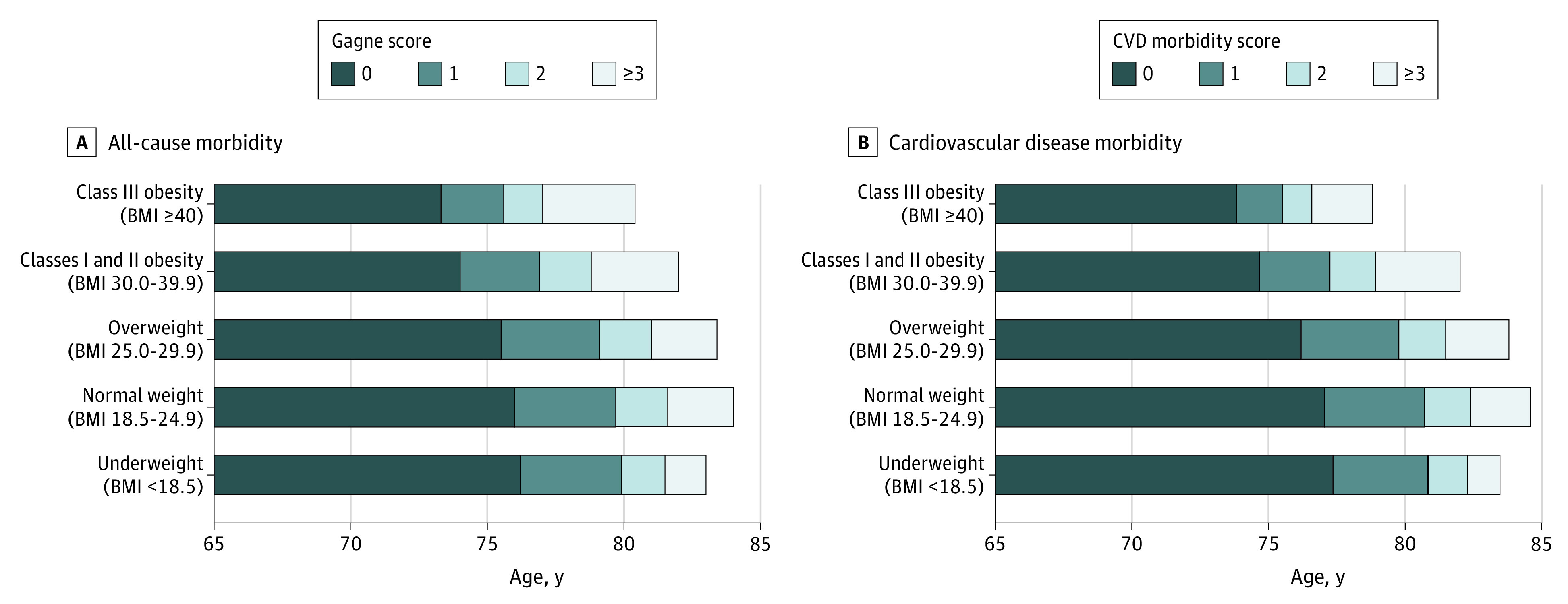
Total Longevity and Years Lived With All-Cause and Cardiovascular Disease (CVD) Morbidity in Older Age (≥65 Years) by Body Mass Index (BMI) Category in Midlife Restricted mean survival time analysis provides the mean time in years spent with a Gagne score of 0 compared with scores of 1 to at least 3 (A) as well as without CVD (CVD morbidity score of 0) and with increasing number of cardiovascular conditions with CVD morbidity scores of 1 to at least 3 (B) in older age. For all-cause morbidity, the score represents the Gagne combined comorbidity score (n = 22 058). For CVD morbidity, the score represents a score of cardiovascular conditions (n = 22 763). Body mass index is calculated as weight in kilograms divided by height in meters squared. Total longevity and years lived were adjusted for age, sex, race and ethnicity, educational level, smoking, hypertension, hyperlipidemia, and diabetes.

Years of life lived with a Gagne morbidity score of 0 or without any CVD morbidity (CVD morbidity score of 0) was estimated by BMI category. After adjustment for demographic and risk factors, years lived after age 65 years with a Gagne score of 0 was 11.0 (95% CI, 10.8-11.2) in the normal BMI group, 10.5 (95% CI, 10.3-10.8) in the overweight group, and 9.0 (95% CI, 8.6-9.3) in those with classes I and II obesity (Figure 2; eTable 6 in the Supplement). Specifically, individuals in the overweight category lived, on average, 0.5 or 1 year fewer with a Gagne or CVD morbidity score of 0, respectively. Those in the classes I and II obesity category lived, on average, 2.0 or 2.7 years fewer with a Gagne or CVD morbidity score of 0, respectively. Similar patterns were observed with the Charlson morbidity score of 0, 1, 2, and 3 or higher (eTable 7 in the Supplement).

During follow-up, 13 932 participants (47.0%) died, with a similar mean age at death among participants in the overweight category (82.1 years [95% CI, 81.9-82.2 years]) and younger for those in the classes I and II obesity (80.8 years [95% CI, 80.5-81.1 years]) and class III obesity (77.7 years [95% CI, 76.2-79.1 years]) categories compared with participants in the normal BMI category (82.3 years [95% CI, 82.1-82.5 years]; eTable 8 in the Supplement). The proportion (SE) of life-years lived in older adulthood with Gagne score of at least 1 was 0.38% (0.00%) in those with a normal BMI, 0.41% (0.00%) in those with overweight, and 0.43% (0.01%) in those with obesity. Consistent findings were observed in mean years of life lived free of morbidity, mean age at death, and proportion of life after age 65 years lived, with CVD morbidity based on a CVD score summing individual CVD conditions. In secondary analyses stratified by sex, similar patterns were observed (eFigure 6 and eTable 8 in the Supplement).

### Medicare Costs in Older Adulthood by BMI in Midlife

Cumulative and mean annual Medicare costs per person after age 65 years by midlife BMI category are provided in Table 2. Relative to those with a normal midlife BMI, cumulative excess costs after age 65 years were higher for individuals who were overweight in midlife by a median of $12 390 (95% CI, $10 427-$14 354) and for individuals who had classes I and II obesity in midlife by a median of $23 396 (95% CI, $18 474-$28 319). Similar differences were observed for cumulative costs at the 25th, 75th, and 90th percentiles as well as excess mean annualized costs after age 65 years at the 25th, 50th, 75th, and 90th percentiles. Patterns for both cumulative and mean annual costs were similar, with consistently higher costs for midlife overweight and obesity in sensitivity analyses when the analysis included those who had Medicare data that began after age 65 years (eTable 9 in the Supplement), was stratified by sex (eTables 10 and 11 in the Supplement), and excluded those with zero costs (eTable 12 in the Supplement).

**Table 2.** Differences in Cumulative Cost and Average Annual Cost in Older Age Stratified by BMI Category in Midlife Among the 23 342 Study Participants^a^

BMI category	Quantile regression coefficient by percentile				
	25th	50th	75th	90th	
**Cumulative cost (95% CI), $** ^b^					
Underweight, (BMI, <18.5)	0 (−4 to 4)	−3303 (−4231 to −2376)	−22 840 (−27 585 to −18 096)	−87 718 (−109 694 to −65 741)	
Overweight (BMI, 25.0-29.9)	147 (126 to 167)	12 390 (10 427 to 14 354)	36 675 (30 316 to 43 035)	57 100 (45 080 to 69 121)	
Classes I and II obesity (BMI, 30.0-39.9)	501 (443 to 559)	23 396 (18 474 to 28 319)	65 492 (52 985 to 78 000)	108 884 (85 735 to 132 032)	
Class III obesity (BMI, ≥40)	0 (−412 to 412)	10 788 (−23 130 to 44 705)	72 640 (1548 to 143 733)	75 500 (−96 515 to 247 516)	
**Mean annual cost (95% CI), $** ^c^					
Underweight, (BMI, <18.5)	0 (0 to 0)	−137 (−233 to −40)	−1725 (−2426 to −1024)		−3801 (−6707 to −894)
Overweight (BMI, 25.0-29.9)	18 (16 to 19)	911 (735 to 1088)	1874 (1389 to 2360)		2862 (1754 to 3970)
Classes I and II obesity (BMI, 30.0-39.9)	58 (53 to 64)	2048 (1578 to 2517)	4842 (3808 to 5876)		7881 (5541 to 10 221)
Class III obesity (BMI, ≥40)	−34 (−64 to −3)	2109 (−1333 to 5552)	7451 (−2802 to 17 705)		24 769 (−41 508 to 91 045)

Abbreviation: BMI, body mass index (calculated as weight in kilograms divided by height in meters squared).

^a^ Each column is from a separate regression. Coefficients (cost differences) and 95% CIs (in parentheses) are reported. All regressions also adjust for baseline age, race and ethnicity, sex, educational level, whether the individual had diabetes, hyperlipidemia, or hypertension at baseline, whether the individual smoked at baseline, and death. Costs are in 2016 US dollars.

^b^ Data represent comparison of each BMI category with the normal category (BMI, 18.5-24.9) (reference group). For individuals in the the reference group, cumulative costs at the 25th, 50th, 75th, and 90th percentiles were −$382, $2924, $30 868, and $99 040, respectively.

^c^ Data represent comparison of each BMI category with the normal category (reference group). For the reference group, mean annual costs at the 25th, 50th, 75th, and 90th percentiles were −$34, $483, $3728, and $8482, respectively.

## Discussion

In this cohort study with long-term follow-up that used available Medicare data from 1985 to 2015, we found individuals who were overweight or had classes I and II obesity in midlife (mean age 40 years at baseline) had significantly higher cumulative overall and CVD morbidity burden. Similar findings were observed with 2 validated morbidity indices as well as with summing of individual CVD conditions. The higher morbidity burden was observed in the context of similar longevity among participants in the normal BMI and overweight categories and shorter longevity among those in the classes I and II obesity and class III obesity categories. The greater proportion of life lived with morbidity translated to higher adjusted cumulative and mean annual health care expenditures during older adulthood, which will have substantial consequences for health care costs as the aging population intersects with the obesity epidemic.

Our findings provide evidence that overweight or obesity status decades earlier, in midlife, is independently associated with a greater cumulative burden of morbidity in older adulthood as assessed with the Gagne morbidity score and when summing CVD conditions for every year after age 65 years. These data expand on prior reports from a smaller subset of the CHA, which demonstrated an association between higher BMI and greater risk for CVD hospitalization as well as higher Medicare costs through 2002.^5,21,22,23,24^ Our findings extend this work with a broader proportion of the CHA cohort and longer follow-up through age 90 years or death. We additionally quantified average longevity, which contextualizes the differences between morbidity and life span. Our data demonstrate the higher burden of morbidity despite similar total life span among individuals in the overweight category as well as a higher burden of morbidity and a shorter total life span among those in the classes I and II obesity and class III obesity categories. While prior work has identified an overweight or obesity “paradox” whereby individuals with obesity who have pre-existing CVD or long-standing end-organ dysfunction, such as chronic obstructive pulmonary disease, chronic kidney disease, and cirrhosis,^25^ have longer survival relative to those with normal BMI, this may be owing to unintentional weight loss, reflecting greater severity of illness or frailty, sarcopenia, or an increased catabolic state in the reference group (normal BMI) once end-organ disease is present. Our work suggests that there is not a favorable survival advantage for individuals in high-BMI categories, such as overweight or obesity.

### Strengths and Limitations

Strengths of our study include the large size of the CHA cohort, the long-term follow-up, and the objective measurement of BMI early in the life course. Our findings support a broader emphasis on maintaining an optimal body weight, beginning early in adulthood. Health promotion efforts require an expanded focus to also target the overweight BMI category to successfully reduce preventable morbidity and health care costs. Earlier age at onset of overweight and obesity is especially concerning because cumulative exposure to adverse BMI levels or excess weight has been associated with a higher risk of subclinical^26,27,28^ and clinical^29^ CVD. According to estimates from the National Health and Nutrition Examination Surveys 2015 to 2018, age-adjusted prevalence of overweight and obesity is 35.4% in youths.^30^ These numbers are anticipated to increase in the coming decades as these youths become adults, with simulation models estimating that, among all children aged 2 to 19 years in 2016, risk of obesity by age 35 years will be 57.3%.^31^

Important limitations of this work should be noted. First, the sample included in CHA was recruited between 1967 and 1973 and follow-up started in 1985. While secular changes in risk factor prevalence have occurred over the time studied,^32^ follow-up spanning decades is needed to quantify long-term estimates of morbidity. Lack of follow-up between the in-person examination and available Medicare data may have led to underestimation of morbidity burden. Second, the sample was predominantly White and may be healthier than the general population, which may limit the generalizability of the findings in more diverse samples. Third, participants who died before the age of 65 years and those who did not enroll in Medicare (a small number of participants who did not enroll in Medicare [eg, Veterans Administration enrollees]) as well as those with missing covariates were excluded, which may additionally limit the generalizability of the findings. However, these exclusions compose only a small proportion of the total cohort. In addition, there was a similar BMI distribution among those included compared with those excluded. Fourth, use of Medicare administrative claims data has the potential for miscoding or underdiagnosis of morbidity. However, Medicare claims data represent among the most comprehensive and robust data for epidemiologic and health services research in older adults.^33,34^ In addition, integration in morbidity indices to quantify morbidity may lead to an underestimate of total burden, which may bias the findings toward the null. However, the Gagne and Charlson morbidity scores have been well validated for use in administrative data sets.^17,18^ The Gagne score was developed as a multivariable estimation of mortality, which weights more heavily morbidity that is more strongly associated with mortality and therefore may underestimate morbidity burden distinct from mortality. However, we note similar findings with an alternate overall morbidity score (the Charlson Comorbidity Index) and a CVD morbidity score that represents a sum score of any CVD condition similar to prior publications,^35^ which supports the primary findings. Fifth, given that morbidity may develop before age 65 years, estimation of time lived with morbidity limited to older adulthood is likely underestimating time with morbidity and biases our findings toward the null. However, in a sensitivity analysis that included all participants (including those with morbidity at age 65 years), we observed similar patterns. Future cohort studies that assess participants from young adulthood to midlife prior to Medicare enrollment should be pursued to address this important limitation. Sixth, results for participants in the underweight and class III obesity categories should be interpreted with caution because these subgroups were very small. Additionally, there is the possibility that healthy survival bias at the extremes of BMI may explain, in part, the observed findings in the class III obesity group. Seventh, the lack of repeated measures of BMI over time limits our ability to account for changes over time because many younger adults who are overweight may transition to being obese. However, our results underscore the importance of assessing and optimizing BMI early in life.

## Conclusions

In this cohort study, the findings indicate that higher BMI in midlife in the overweight category was associated with a greater burden of overall and CVD morbidity in the context of a similar longevity compared with individuals with a normal BMI. This association also translated to higher health care costs in older adulthood. Resources and strategies are urgently needed at the individual and population level to address the growing public health challenge of excess weight in the context of an aging population.

## Notes

Supplement. **eFigure 1.** Flowchart for Analyzed Sample From the Chicago Heart Association Detection Project in Industry **eFigure 2.** Mean Morbidity Score (Charlson Comorbidity Index) in Older Age by Body Mass Index Category in Midlife Among Those With a Morbidity Score of 0 at Age 65 Years **eFigure 3.** Mean Gagne (A) and Cardiovascular (B) Morbidity Scores in Older Age by Body Mass Index Category in Midlife **eFigure 4.** Mean Gagne (A) and Cardiovascular (B) Morbidity Scores in Older Age by Body Mass Index Category in Midlife Among Those With a Morbidity Score of 0 at Age 65 Years Stratified by Sex **eFigure 5.** Mean Gagne (A) and Cardiovascular Disease (B) Morbidity in Older Age in Men and Women by Body Mass Index Category in Midlife **eFigure 6.** Average Years Lived with Gagne (A) and Cardiovascular (B) Morbidity Scores of 0, 1, 2, and 3+ in Older Age (≥65 Years) in Men and Women by Body Mass Index Category in Midlife **eTable 1.** Distribution of Body Mass Index in Included vs Excluded Individuals **eTable 2.** Gagne Combined Comorbidity Score Conditions and Weights Derived From Medicare Population **eTable 3.** Baseline Demographic Characteristics and Risk Factors by Body Mass Index Category in Midlife, Analytic Dataset **eTable 4.** Included Sample Size Through Average Age, 90 Years, Stratified by Body Mass Index in Midlife **eTable 5.** Area Under the Curve for Cumulative Gagne Morbidity Score, Cardiovascular Disease Morbidity Score, and Charlson Morbidity Score Overall and Stratified by Sex **eTable 6.** Adjusted Years Lived With Gagne and Cardiovascular Morbidity Scores of 0, 1, 2, and 3 or More Stratified by Body Mass Index Category in Midlife **eTable 7.** Adjusted Years Lived with Charlson Comorbidity Index of 0, 1, 2, 3+ Stratified by Body Mass Index Category in Midlife **eTable 8.** Average Age of Morbidity Incidence, Death, and the Proportion of Life Years Lived With Morbidity Overall and Stratified by Sex **eTable 9.** Differences in Cumulative Cost in Older Age by Body Mass Index Category in Midlife Stratified by Sex **eTable 10.** Differences in Average Annual Cost in Older Age by Body Mass Index Category in Midlife Stratified by Sex **eTable 11.** Differences in Cumulative Cost and Average Annual Cost in Older Age by Body Mass Index Category in Midlife in All Participants With Available Medicare Data **eTable 12.** Differences in Cumulative Cost and Average Annual Cost in Older Age by Body Mass Index Category in Midlife Excluding Individuals With Zero Costs
