# Supplementary material for: Association of Body Mass Index in Midlife With Morbidity Burden in Older Adulthood and Longevity
Source: JAMA Netw Open. 2022 Mar 15;5(3):e222318. doi: 10.1001/jamanetworkopen.2022.2318 (PMC8924714; doi:10.1001/jamanetworkopen.2022.2318)
Supplement: Supplement. — eFigure 1. Flowchart for Analyzed Sample From the Chicago Heart Association Detection Project in Industry eFigure 2. Mean Morbidity Score (Charlson Comorbidity Index) in Older Age by Body Mass Index Category in Midlife Among Those With a Morbidity Score of 0 at Age 65 Years eFigure 3. Mean Gagne (A) and Cardiovascular (B) Morbidity Scores in Older Age by Body Mass Index Category in Midlife eFigure 4. Mean Gagne (A) and Cardiovascular (B) Morbidity Scores in Older Age by Body Mass Index Category in Midlife Among Those With a Morbidity Score of 0 at Age 65 Years Stratified by Sex eFigure 5. Mean Gagne (A) and Cardiovascular Disease (B) Morbidity in Older Age in Men and Women by Body Mass Index Category in Midlife eFigure 6. Average Years Lived with Gagne (A) and Cardiovascular (B) Morbidity Scores of 0, 1, 2, and 3+ in Older Age (≥65 Years) in Men and Women by Body Mass Index Category in Midlife eTable 1. Distribution of Body Mass Index in Included vs Excluded Individuals eTable 2. Gagne Combined Comorbidity Score Conditions and Weights Derived From Medicare Population eTable 3. Baseline Demographic Characteristics and Risk Factors by Body Mass Index Category in Midlife, Analytic Dataset eTable 4. Included Sample Size Through Average Age, 90 Years, Stratified by Body Mass Index in Midlife eTable 5. Area Under the Curve for Cumulative Gagne Morbidity Score, Cardiovascular Disease Morbidity Score, and Charlson Morbidity Score Overall and Stratified by Sex eTable 6. Adjusted Years Lived With Gagne and Cardiovascular Morbidity Scores of 0, 1, 2, and 3 or More Stratified by Body Mass Index Category in Midlife eTable 7. Adjusted Years Lived with Charlson Comorbidity Index of 0, 1, 2, 3+ Stratified by Body Mass Index Category in Midlife eTable 8. Average Age of Morbidity Incidence, Death, and the Proportion of Life Years Lived With Morbidity Overall and Stratified by Sex eTable 9. Differences in Cumulative Cost in Older Age by Body Mass Index Category in Midlife Stratified b [file jamanetwopen-e222318-s001.pdf]

Supplementary Online Content

Khan SS, Krefman AE, Zhao L, et al. Association of body mass index in midlife with morbidity burden in older adulthood and longevity. *JAMA Netw Open*. 2022;5(3):e222318.
doi:10.1001/jamanetworkopen.2022.2318

eFigure 1. Flowchart for Analyzed Sample from the Chicago Heart Association Detection Project in Industry

eFigure 2. Mean Morbidity Score (Charlson Comorbidity Index) in Older Age by Body Mass Index Category in Midlife Among Those With a Morbidity Score of 0 at Age 65 Years

eFigure 3. Mean Gagne (A) and Cardiovascular (B) Morbidity Scores in Older Age by Body Mass Index Category in Midlife

eFigure 4. Mean Gagne (A) and Cardiovascular (B) Morbidity Scores in Older Age by Body Mass Index Category in Midlife Among Those With a Morbidity Score of 0 at Age 65 Years Stratified by Sex

eFigure 5. Mean Gagne (A) and Cardiovascular (B) Morbidity in Older Age in Men and Women by Body Mass Index Category in Midlife

eFigure 6. Average Years Lived With Gagne (A) and Cardiovascular (B) Morbidity Scores of 0, 1, 2, and 3+ in Older Age (≥ 65 Years) in Men and Women by Body Mass Index Category in Midlife

eTable 1. Distribution of Body Mass Index in Included vs Excluded Individuals

eTable 2. Gagne Combined Comorbidity Score Conditions and Weights Derived From Medicare Population

eTable 3. Baseline Demographic Characteristics and Risk Factors by Body Mass Index Category in Midlife, Analytic Dataset

eTable 4. Included Sample Size Through Average Age, 90 Years, Stratified by Body Mass Index in Midlife

eTable 5. Area Under the Curve for Cumulative Gagne Morbidity Score, Cardiovascular Disease Morbidity Score, and Charlson Morbidity Score Overall and Stratified by Sex

eTable 6. Adjusted Years Lived With Gagne and Cardiovascular Morbidity Scores of 0, 1, 2, and 3 or More Stratified by Body Mass Index Category in Midlife

eTable 7. Adjusted Years Lived With Charlson Comorbidity Index of 0, 1, 2, 3+ Stratified by Body Mass Index Category in Midlife

eTable 8. Average Age of Morbidity Incidence, Death, and the Proportion of Life Years Lived With Morbidity Overall and Stratified by Sex

eTable 9. Differences in Cumulative Cost in Older Age by Body Mass Index Category in Midlife Stratified by Sex

eTable 10. Differences in Average Annual Cost in Older Age by Body Mass Index Category in Midlife Stratified by Sex

eTable 11. Differences in Cumulative Cost and Average Annual Cost in Older Age by Body Mass Index Category in Midlife in All Participants With Available Medicare Data

eTable 12. Differences in Cumulative Cost and Average Annual Cost in Older Age by Body Mass Index Category in Midlife Excluding Individuals With Zero Costs

This supplementary material has been provided by the authors to give readers additional information about their work.

© 2022 Khan SS et al. *JAMA Network Open.*

eFigure 1. Flowchart for Analyzed Sample from the Chicago Heart Association Detection Project in Industry

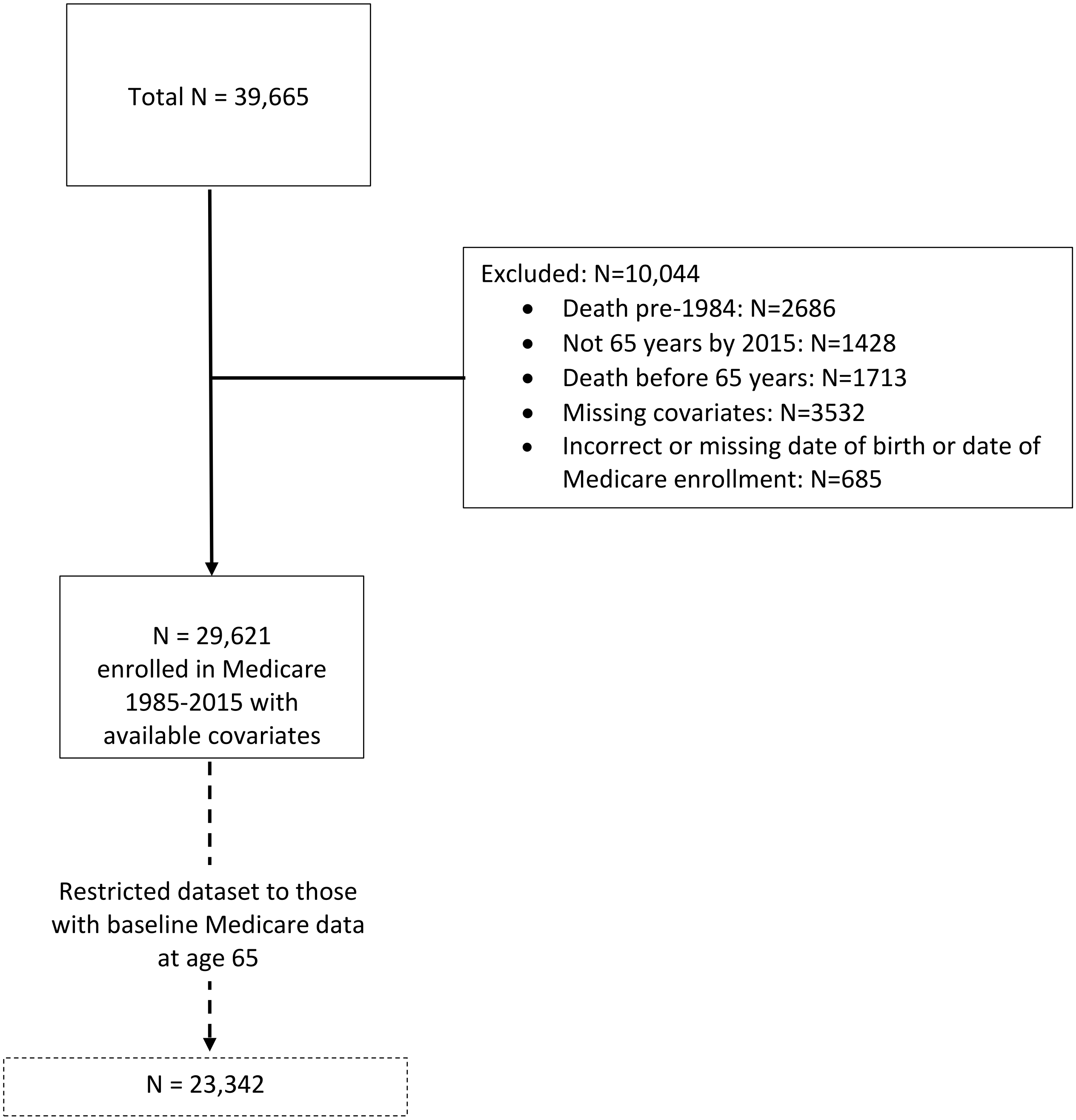

© 2022 Khan SS et al. *JAMA Network Open.*

eFigure 2. Mean Morbidity Score (Charlson Comorbidity Index) in Older Age by Body Mass Index Category in Midlife Among Those With a Morbidity Score of 0 at Age 65 Years, N=22,382

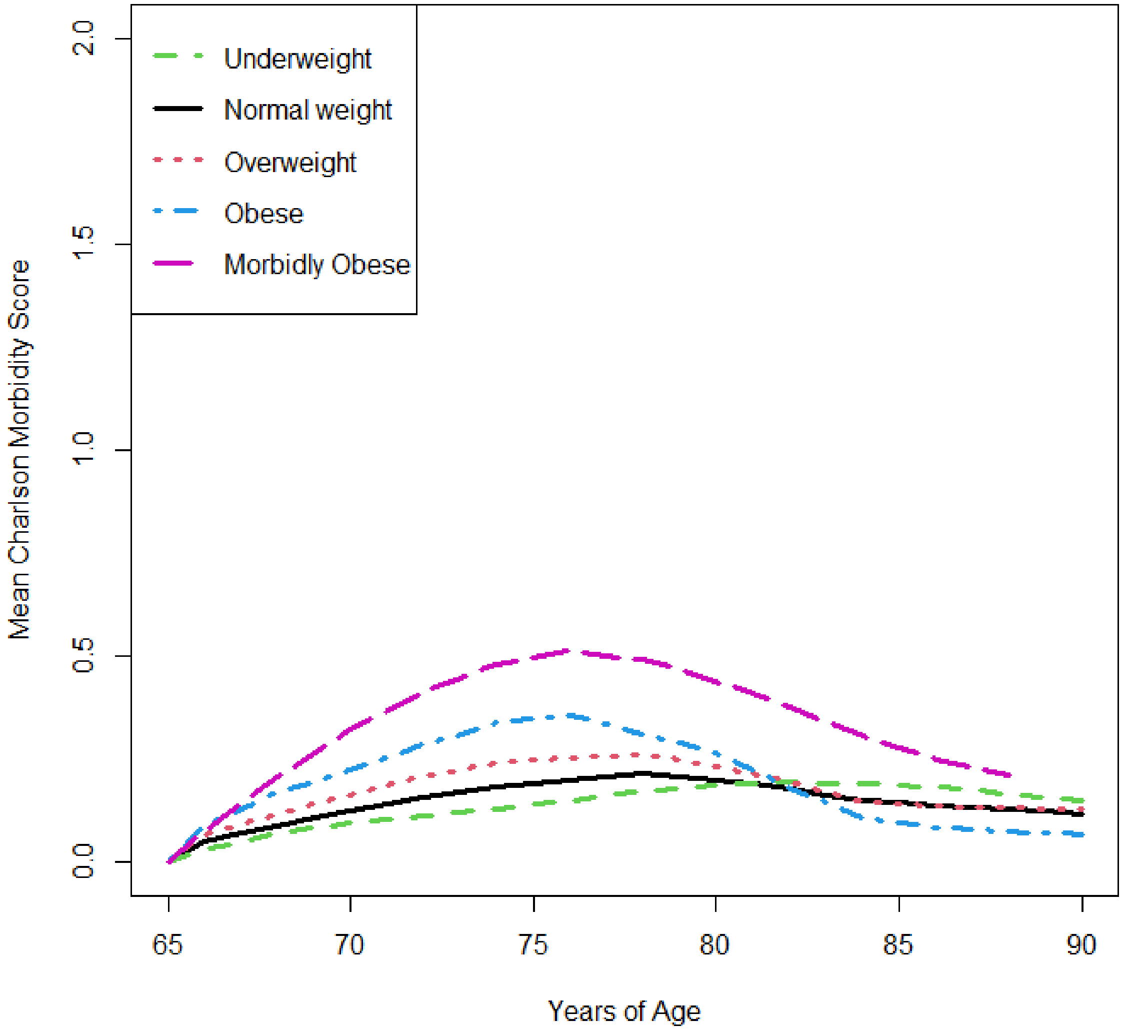

© 2022 Khan SS et al. *JAMA Network Open.*

eFigure 3. Mean Gagne (A) and Cardiovascular (B) Morbidity Scores in Older Age by Body Mass Index Category in Midlife, N=23,342

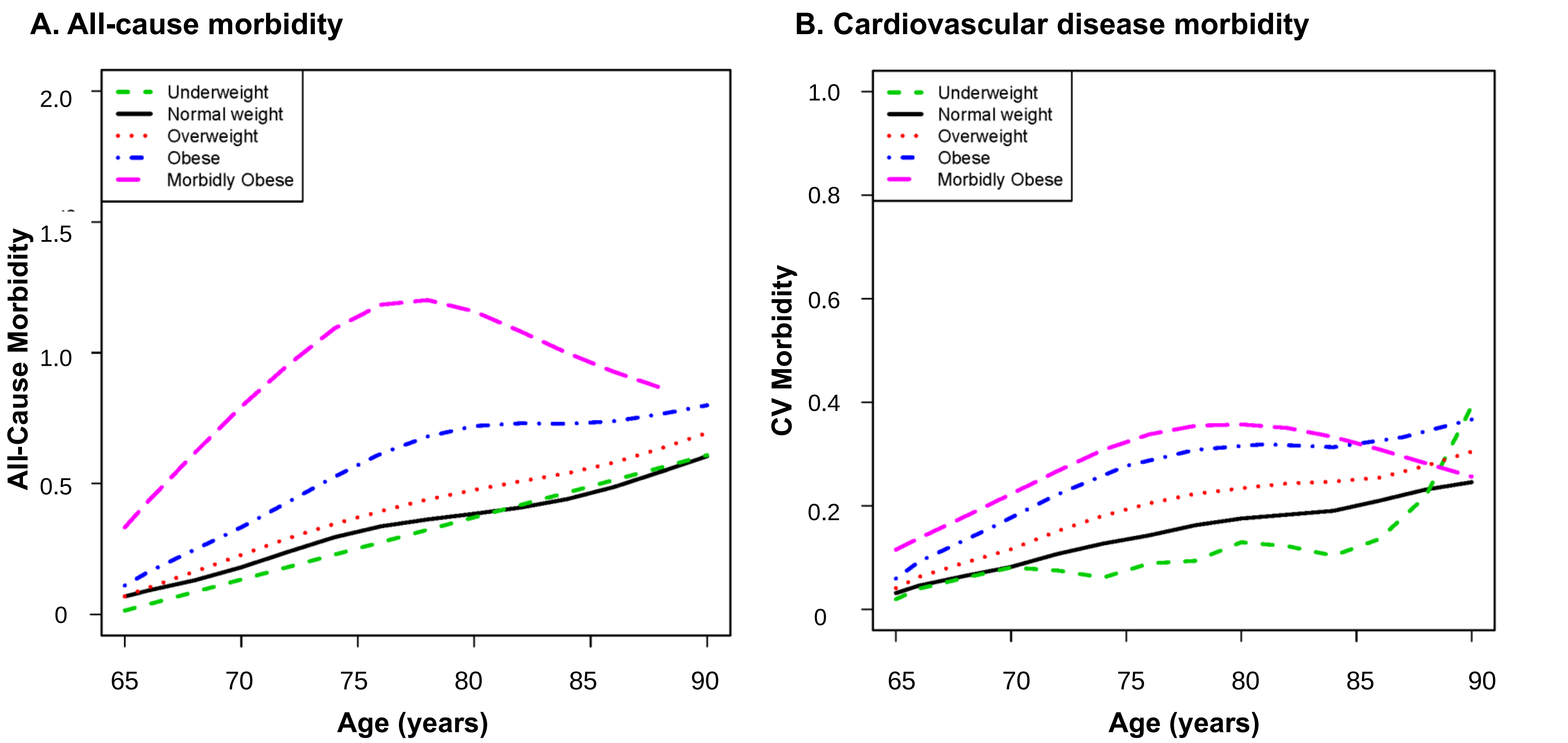

© 2022 Khan SS et al. *JAMA Network Open.*

eFigure 4. Mean Gagne (A) and Cardiovascular (B) Morbidity Scores in Older Age by Body Mass Index Category in Midlife Among Those With a Morbidity Score of 0 at Age 65 Years Stratified by Sex

A. Men, Gagne Morbidity (N=12,949)

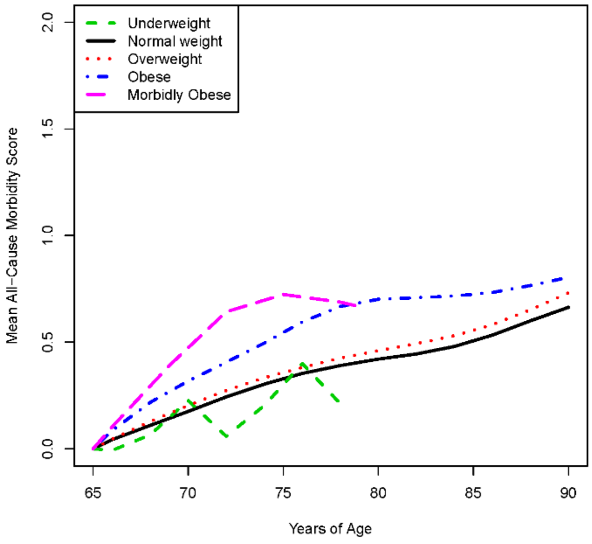

B. Men, CVD Morbidity (N=13,367)

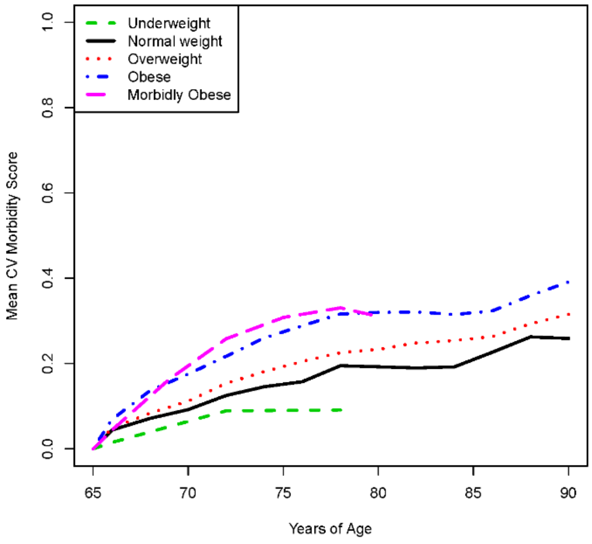

C. Women, Gagne Morbidity (N=9,109)

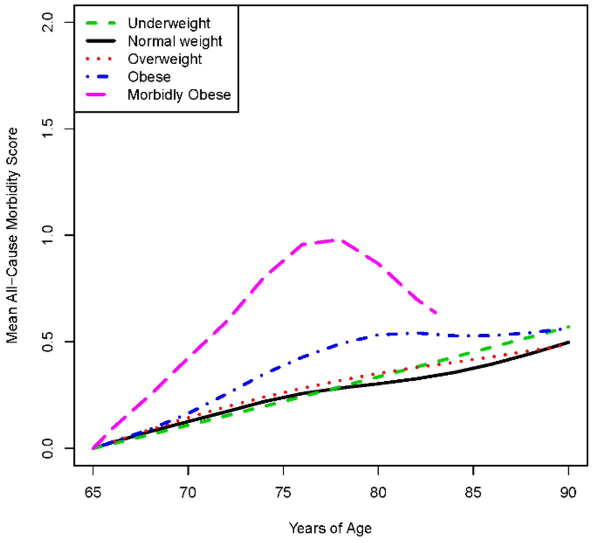

D. Women, CVD Morbidity (N=9,396)

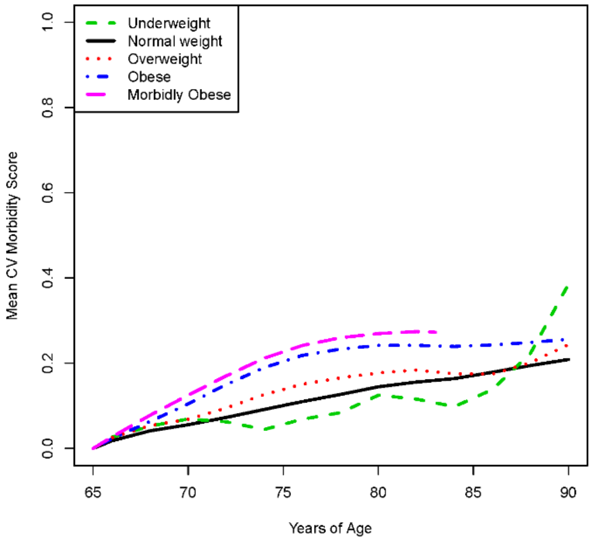

© 2022 Khan SS et al. *JAMA Network Open.*

eFigure 5. Mean Gagne (A) and Cardiovascular (B) Morbidity in Older Age in Men and Women by Body Mass Index Category in Midlife

A. Men, Gagne Morbidity (N=13,761)

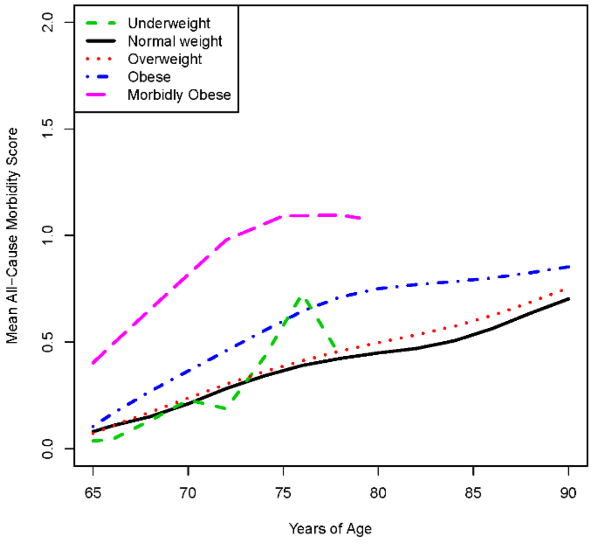

B. Men, CVD Morbidity (N=13,761)

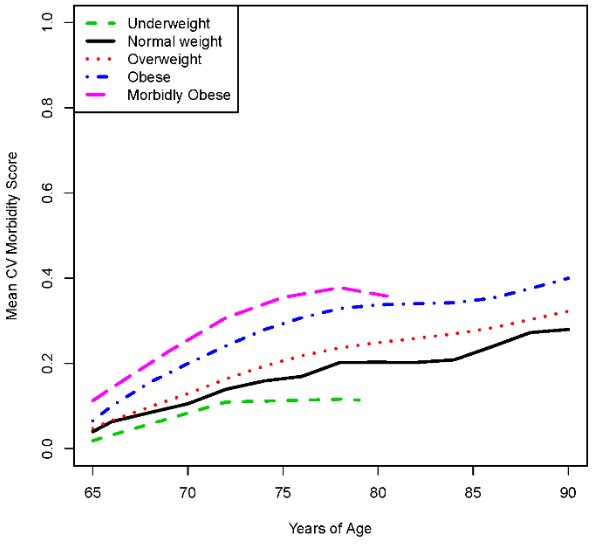

C. Women, Gagne Morbidity (N=9,581)

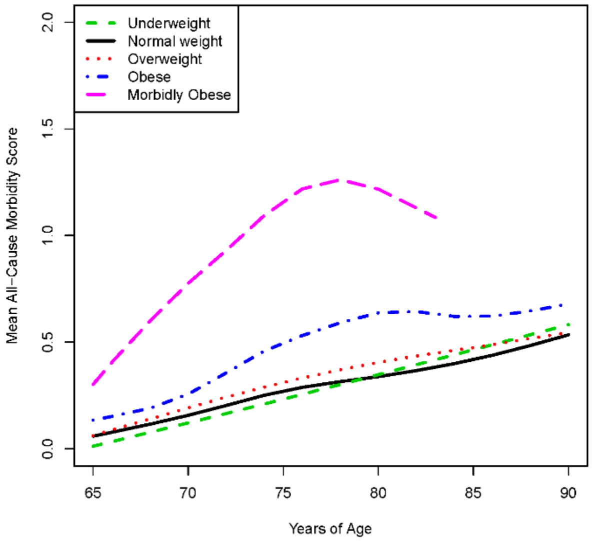

D. Women, CVD Morbidity (N=9,581)

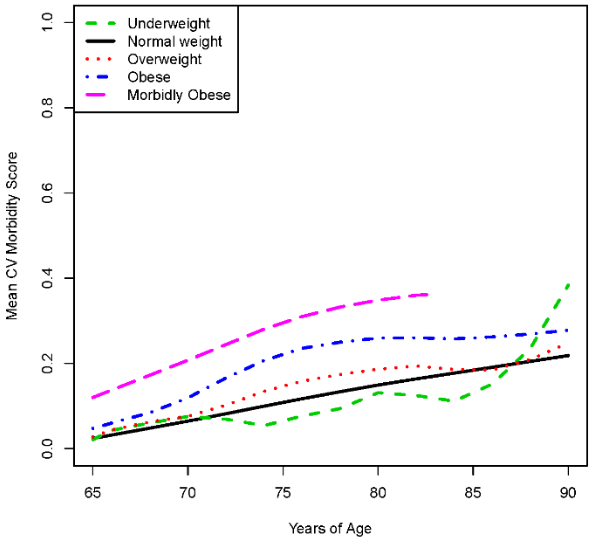

© 2022 Khan SS et al. *JAMA Network Open.*

eFigure 6. Average Years Lived With Gagne (A) and Cardiovascular (B) Morbidity Scores of 0, 1, 2, and 3+ in Older Age (≥ 65 years) in Men and Women by Body Mass Index Category in Midlife

A. Men, Gagne Morbidity (N=12,949)

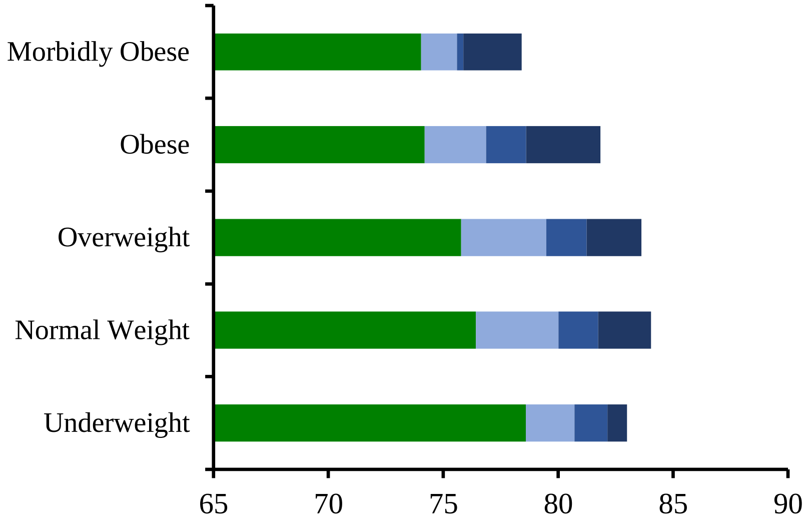

B. Men, CVD Morbidity (N=13,367)

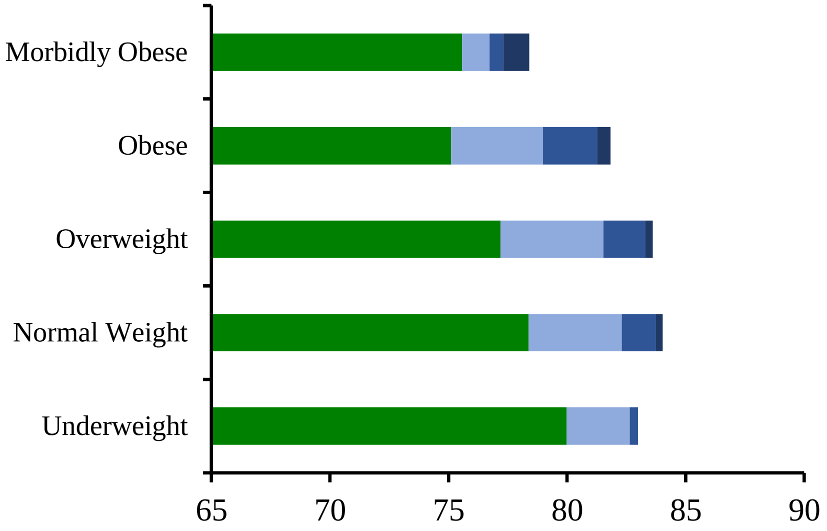

C. Women, All-Cause Morbidity (N=9,109)

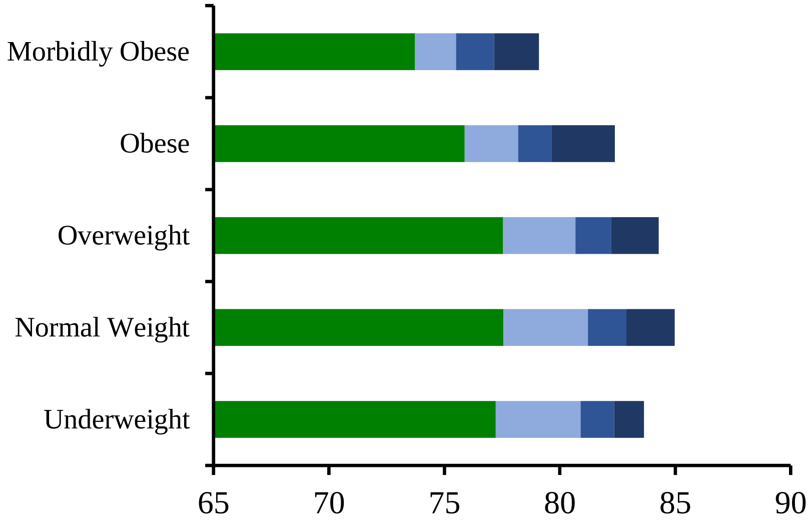

D. Women, CVD Morbidity (N=9,396)

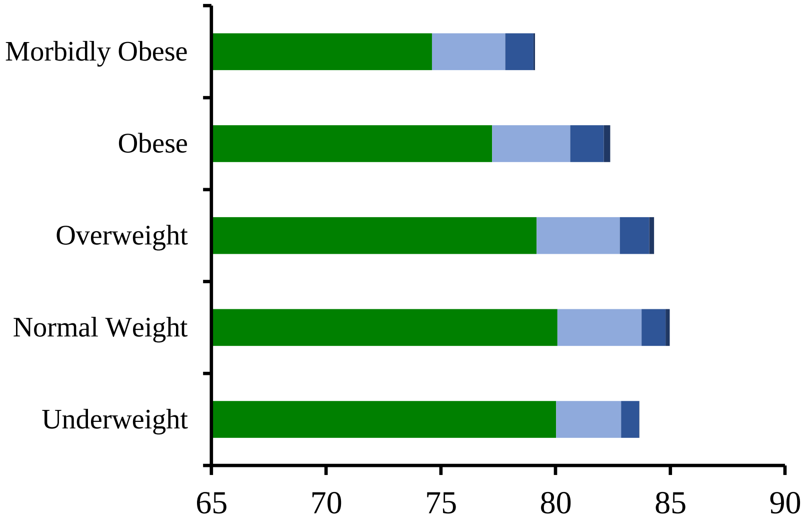

© 2022 Khan SS et al. *JAMA Network Open.*

eTable 1. Distribution of Body Mass Index in Included vs Excluded Individuals

**BMI Category**	**Included** N=29,621	**Excluded** N=10,044
Underweight, (BMI<18.5 kg/m^2)	600 (2)	331 (3.3)
Normal BMI, (18.5-24.9 kg/m^2)	13638 (46)	4541 (45.2)
Overweight, (BMI 25.0-29.9 kg/m^2)	11743 (39.6)	3657 (36.4)
Obese (BMI 30.0-39.9 kg/m^2)	3515 (11.9)	1440 (14.3)
Morbidly obese (BMI ≥ 40 kg/m^2)	125 (0.4)	75 (0.8)

© 2022 Khan SS et al. *JAMA Network Open.*

eTable 2. Gagne Combined Comorbidity Score Conditions and Weights Derived From Medicare Population

**Condition**	**Weight**
Metastatic cancer	5
Heart failure	2
Dementia	2
Renal failure	2
Weight loss	2
Hemiplegia	1
Alcohol abuse	1
Any tumor	1
Cardiac arrhythmia	1
Chronic pulmonary disease	1
Coagulopathy	1
Complicated diabetes	1
Deficiency anemias	1
Fluid and electrolyte disorders	1
Liver disease	1
Peripheral vascular disorder	1
Psychosis	1
Pulmonary circulation disorders	1
HIV/AIDS	-1
Hypertension	-1

© 2022 Khan SS et al. *JAMA Network Open.*

eTable 3. Baseline Demographic Characteristics and Risk Factors by Body Mass Index Category in Midlife, Analytic Dataset

	**Underweight**<br><br>**BMI**<br>**<18.5**	**Normal**<br><br>**BMI**<br>**18.5-24.9**	**Overweight**<br><br>**BMI**<br>**25-29.9**	**Obesity**<br><br>**BMI**<br>**30-39.9**	**Morbid Obesity**<br>**BMI**<br>**≥40**	**p-value**
	**N=545**	**N=11,220**	**N=8,935**	**N=2540**	**N=102**	
**Demographic Characteristics**						
Mean age at baseline (SD)	28.7 (8.5)	33.6 (9.5)	36.9 (8.8)	38.1 (8.8)	38.4 (9.7)	<0.001
Female, n (%)	56 (10.3)	4897 (43.6)	6925 (77.5)	1841 (72.5)	42 (41.2)	<0.001
Male, n (%)	489 (89.7)	6323 (56.4)	2010 (22.5)	699 (27.5)	60 (58.8)	
Black, n (%)	118 (21.7)	1339 (11.9)	740 (8.3)	272 (10.7)	18 (17.6)	<0.001
Education						
<=High school	298 (54.7)	5965 (53.2)	4458 (49.9)	1536 (60.5)	68 (66.7)	<0.001
Some college	122 (22.4)	2246 (20.0)	1723 (19.3)	463 (18.2)	24 (23.5)	
College graduate	125 (22.9)	3009 (26.8)	2754 (30.8)	541 (21.3)	10 (9.8)	
**Risk Factors**						
Diabetes, n (%)	5 (0.9)	155 (1.4)	105 (1.2)	63 (2.5)	3 (2.9)	<0.001
Current smoking, n (%)	265 (48.6)	5000 (44.6)	3606 (40.4)	980 (38.6)	43 (42.2)	<0.001
Mean systolic blood pressure, mm Hg	119.4 (13.4)	127.3 (14.8)	135.3 (16.2)	142.2 (17.8)	159.0 (23.6)	<0.001
Hypertension, n (%)	61 (11.2)	3167 (28.2)	4386 (49.1)	1672 (65.8)	94 (92.2)	<0.001
Mean total cholesterol, mg/dL	174.7 (33.2)	189.3 (36.3)	202.1 (36.8)	206.2 (37.7)	200.0 (40.7)	<0.001
Dyslipidemia, n (%)	19 (3.5)	994 (8.9)	1338 (15.0)	488 (19.2)	17 (16.7)	<0.001

BMI, body mass index (calculated as weight in kilograms divided by height in meters squared)

© 2022 Khan SS et al. *JAMA Network Open.*

eTable 4. Included Sample Size Through Average Age, 90 Years, Stratified by Body Mass Index in Midlife

	**65 years**	**70 years**	**75 years**	**80 years**	**85 years**	**90 years**
Underweight, N	545	277	146	85	34	12
Normal BMI, N	11220	8194	5662	3799	2291	947
Overweight, N	8935	7582	5658	3885	2228	879
Obesity, N	2540	2135	1565	1062	594	202
Morbid obesity, N	102	82	49	31	19	12
**Overall, N**	**23342**	**18270**	**13080**	**8862**	**5166**	**2052**

© 2022 Khan SS et al. *JAMA Network Open.*

eTable 5. Area Under the Curve for Cumulative Gagne Morbidity Score, Cardiovascular Disease Morbidity Score, and Charlson Morbidity Score Overall and Stratified by Sex

	**Gagne Morbidity N=22,058**		**CVD Morbidity N=22,763**		**Charlson Morbidity Index N=22,382**	
	AUC	Adjusted p-value	AUC	Adjusted p-value	AUC	Adjusted p-value
**Overall**						
Underweight	5.78	0.15	2.12	<.001	3.99	0.95
Normal BMI	6.10	Ref.	2.88	Ref.	3.97	Ref.
Overweight	7.22	<.001	3.84	<.001	4.62	<.001
Obesity	9.80	<.001	5.02	<.001	5.24	<.001
Morbid obesity	10.32	<.001	4.15	<.001	6.93	<.001
**Men**						
Underweight	5.34	<.001	1.63	<.001	1.98	<.001
Normal BMI	7.63	Ref.	3.40	Ref.	3.57	Ref.
Overweight	8.02	<.001	4.14	<.001	4.60	<.001
Obesity	11.00	<.001	5.48	<.001	5.05	<.001
Morbid obesity	8.03	0.33	4.47	<.001	6.85	<.001
**Women**						
Underweight	5.86	0.002	2.18	0.003	4.27	0.83
Normal BMI	5.12	Ref.	2.50	Ref.	4.20	Ref.
Overweight	5.19	0.34	3.00	<.001	4.74	<.001
Obesity	7.16	<.001	3.96	<.001	5.64	<.001
Morbid obesity	11.75	<.001	3.93	<.001	6.95	<.001

AUC reflects area under the curve; BMI reflects body mass index; CVD reflects cardiovascular disease

© 2022 Khan SS et al. *JAMA Network Open.*

eTable 6. Adjusted Years Lived With Gagne and Cardiovascular Morbidity Scores of 0, 1, 2, and 3 or More Stratified by Body Mass Index Category in Midlife*

**BMI Category**				
**Gagne morbidity score, N=22,058**				
	**Time with Gagne Score=0, yrs**	**Time with Gagne score=1, yrs**	**Time with Gagne Score=2, yrs**	**Time with Gagne score=3+, yrs**
Underweight, (BMI<18.5 kg/m^2)	11.2 (10.1, 12.2)	3.7 (2.9, 4.5)	1.6 (1.0, 2.2)	1.5 (0.8, 2.2)
Normal BMI, (18.5-24.9 kg/m^2)	11.0 (10.8, 11.2)	3.7 (3.6, 3.9)	1.9 (1.7, 2.0)	2.4 (2.2, 2.5)
Overweight, (BMI 25.0-29.9 kg/m^2)	10.5 (10.3, 10.8)	3.6 (3.5, 3.8)	1.9 (1.8, 2.0)	2.4 (2.2, 2.5)
Obese (BMI 30.0-39.9 kg/m^2)	9.0 (8.6, 9.3)	2.9 (2.6, 3.1)	1.9 (1.7, 2.1)	3.2 (2.9, 3.4)
Morbidly obese (BMI ≥ 40 kg/m^2)	8.3 (6.4, 10.1)	2.3 (1.4, 3.2)	1.4 (0.9, 2.0)	3.4 (2.0, 4.9)
**Cardiovascular disease morbidity score, N=22,763**				
	**Time with CVD Score=0, yrs**	**Time with CVD score=1, yrs**	**Time with CVD Score=2, yrs**	**Time with CVD score=3+, yrs**
Underweight, (BMI<18.5 kg/m^2)	13.6 (12.5, 14.8)	3.1 (2.3, 3.9)	1.1 (0.5, 1.6)	0.2 (0.0, 0.4)
Normal BMI, (18.5-24.9 kg/m^2)	13.1 (12.9, 13.3)	4.1 (4.0, 4.3)	1.5 (1.4, 1.6)	0.3 (0.2, 0.3)
Overweight, (BMI 25.0-29.9 kg/m^2)	12.1 (11.9, 12.3)	4.3 (4.1, 4.5)	1.7 (1.6, 1.8)	0.3 (0.3, 0.4)
Obese (BMI 30.0-39.9 kg/m^2)	10.4 (10.0, 10.8)	4.0 (3.7, 4.3)	2.1 (1.9, 2.4)	0.4 (0.3, 0.5)
Morbidly obese (BMI ≥ 40 kg/m^2)	10.3 (8.1, 12.5)	3.0 (2.1, 3.9)	1.6 (0.7, 2.5)	0.6 (0.1, 1.3)

*Adjusted for age, sex, race, education, smoking, hypertension, diabetes, and hyperlipidemia and reflect data visualized in Figure 2 in the main manuscript
BMI reflects body mass index

© 2022 Khan SS et al. *JAMA Network Open.*

eTable 7. Adjusted Years Lived With Charlson Comorbidity Index of 0, 1, 2, 3+ Stratified by Body Mass Index Category in Midlife*

**BMI Category**	**Time with Charlson Score=0, yrs**	**Time with Charlson score=1, yrs**	**Time with Charlson Score=2, yrs**	**Time with Charlson score=3+, yrs**
**Charlson morbidity, N=22,382**				
Underweight, (BMI<18.5 kg/m^2)	15.0 (13.8, 16.1)	1.4 (0.7, 2.1)	1.0 (0.4, 1.5)	0.9 (0.5, 1.4)
Normal BMI, (18.5-24.9 kg/m^2)	14.7 (14.5, 14.9)	2.4 (2.2, 2.5)	0.9 (0.8, 0.9)	1.1 (1, 1.2)
Overweight, (BMI 25.0-29.9 kg/m^2)	14.1 (13.8, 14.3)	2.5 (2.4, 2.7)	0.9 (0.8, 1)	1.1 (1, 1.2)
Obese (BMI 30.0-39.9 kg/m^2)	12.3 (11.9, 12.7)	2.2 (1.9, 2.5)	1.1 (0.9, 1.3)	1.4 (1.2, 1.6)
Morbidly obese (BMI ≥ 40 kg/m^2)	10.6 (8.4, 12.8)	1.5 (0.5, 2.4)	1.7 (0.4, 3)	1.8 (0.7, 2.8)

*Adjusted for age, sex, race, education, smoking, hypertension, diabetes, and hyperlipidemia
BMI reflects body mass index

© 2022 Khan SS et al. *JAMA Network Open.*

eTable 8. Average Age of Morbidity Incidence, Death, and the Proportion of Life Years Lived With Morbidity Overall and Stratified by Sex

	Age when Gagne Score≥1 (95% CI), Years	Age at CVD Score ≥1 (95% CI), Years	Age at Charlson Score≥1 (95% CI), Years	Age at Death (95% CI), Years	Proportion of life with Gagne Score≥1 (SE)	Numbers of Years lived with Gagne Score≥1 (SE)	Proportion of life lived with CVD morbidity (SE)	Numbers of Years lived with CVD morbidity (SE)
**Overall**								
Underweight	72.8 (72.1-73.5)	74.3 (73.2-75.4)	73.1 (72.1-74.1)	79.9 (78.7-81)	0.33 (0.02)	6.11 (0.48)	0.19 (0.02)	3.52 (0.46)
Normal BMI	74.9 (74.8-75)	75.6 (75.4-75.7)	74.5 (74.3-74.6)	82.3 (82.1-82.5)	0.38 (0.00)	7.54 (0.09)	0.27 (0.00)	5.27 (0.08)
Overweight	74.9 (74.8-75.1)	75.2 (75.1-75.4)	74.3 (74.2-74.5)	82.1 (81.9-82.2)	0.41 (0.00)	7.61 (0.08)	0.33 (0.00)	6.16 (0.08)
Obesity	74.6 (74.4-74.9)	74.6 (74.4-74.8)	74.1 (73.9-74.3)	80.8 (80.5-81.1)	0.43 (0.01)	7.35 (0.15)	0.37 (0.01)	6.31 (0.16)
Morbid obesity	73.1 (71.9-74.2)	74.1 (72.7-75.4)	72.9 (71.7-74)	77.7 (76.2-79.1)	0.36 (0.05)	4.94 (0.68)	0.28 (0.04)	3.80 (0.69)
**Men**								
Underweight	72.8 (71-74.7)	73.9 (71.5-76.3)	74.3 (71.5-77.1)	79.2 (76-82.4)	0.25 (0.07)	4.41 (1.42)	0.17 (0.06)	3.02 (1.11)
Normal BMI	73.7 (73.5-73.9)	74.1 (73.9-74.3)	73.3 (73.1-73.6)	80.7 (80.4-81)	0.40 (0.01)	7.62 (0.16)	0.30 (0.01)	5.67 (0.14)
Overweight	74.1 (74-74.3)	74.5 (74.4-74.7)	73.6 (73.5-73.8)	81.2 (81-81.4)	0.42 (0.00)	7.84 (0.10)	0.35 (0.00)	6.44 (0.10)
Obesity	73.7 (73.4-73.9)	73.6 (73.4-73.9)	73.2 (72.9-73.5)	80.1 (79.8-80.4)	0.45 (0.01)	7.65 (0.20)	0.40 (0.01)	6.73 (0.19)
Morbid obesity	72.8 (70.9-74.6)	74 (71.6-76.3)	72.2 (70.2-74.3)	77.2 (74.6-79.7)	0.33 (0.07)	4.37 (0.99)	0.21 (0.06)	2.84 (0.85)
**Women**								
Underweight	72.8 (72-73.6)	74.4 (73.2-75.6)	72.9 (71.8-74)	80 (78.7-81.2)	0.35 (0.03)	6.44 (0.60)	0.20 (0.02)	3.64 (0.49)
Normal BMI	75.8 (75.7-76)	76.8 (76.6-77)	75.3 (75.1-75.5)	83.4 (83.1-83.6)	0.37 (0.01)	7.42 (0.12)	0.25 (0.00)	4.90 (0.09)
Overweight	77.2 (77-77.5)	77.5 (77.2-77.8)	76.2 (75.9-76.5)	84 (83.7-84.3)	0.35 (0.01)	6.74 (0.18)	0.26 (0.01)	5.10 (0.17)
Obesity	76.9 (76.5-77.3)	76.9 (76.4-77.4)	76.1 (75.6-76.5)	82.1 (81.6-82.5)	0.37 (0.02)	6.51 (0.31)	0.30 (0.01)	5.14 (0.26)
Morbid obesity	73.3 (71.7-74.8)	74.2 (72.5-75.8)	73.2 (71.9-74.6)	77.9 (76.2-79.6)	0.38 (0.06)	5.37 (0.90)	0.32 (0.05)	4.49 (0.75)

© 2022 Khan SS et al. *JAMA Network Open.*

eTable 9. Differences in Cumulative Cost in Older Age by Body Mass Index Category in Midlife Stratified by Sex, N=23,342

	**Quantile Regression**			
	**25th %**	**50th %**	**75th %**	**90th %**
**Men, N=13,761**	**Cumulative Costs, $ (95% Confidence Interval)**			
	N=13,761	N=13,761	N=13,761	N=13,761
Underweight, (BMI<18.5 kg/m^2)	0 (-1906, 1906)	-1,639 (-5641, 2363)	-19,277 (-36664, -1890)	-61,090 (-215059, 92878)
Normal BMI, (18.5-24.9 kg/m^2)	REFERENT			
Overweight, (BMI 25.0-29.9 kg/m^2)	308 (171, 445)	10,499 (8378, 12620)	33,456 (25587, 41325)	55,126 (41379, 68872)
Obese (BMI 30.0-39.9 kg/m^2)	506 (96, 917)	20,279 (14554, 26003)	55,183 (40966, 69399)	94,422 (69109, 119735)
Morbidly obese (BMI ≥ 40 kg/m^2)	-303 (-2545, 1940)	-1,934 (-27009, 23140)	7,186 (-112454, 126826)	-999 (-184195, 182197)
**Women, N=9,581**	**Cumulative Costs, $ (95% Confidence Interval)**			
	N=9581	N=9581	N=9581	N=9581
Underweight, (BMI<18.5 kg/m^2)	0 (-2, 2)	-2782 (-3966, -1599)	-21,871 (-29156, -14586)	-83,078 (-108582, -57574)
Normal BMI, (18.5-24.9 kg/m^2)	REFERENT			
Overweight, (BMI 25.0-29.9 kg/m^2)	0 (-3, 3)	11,821 (7008, 16634)	40,589 (28707, 52472)	51,031 (31411, 70650)
Obese (BMI 30.0-39.9 kg/m^2)	110 (52, 167)	26,277 (15281, 37273)	86,661 (54376, 118946)	150,983 (107263, 194702)
Morbidly obese (BMI ≥ 40 kg/m^2)	392 (-141, 925)	36,514 (-26910, 99939)	83,070 (-5771, 171912)	105,221 (-175955, 386397)

Notes: Each column is from a separate regression. Coefficients (cost differences) and 95% confidence intervals (in parentheses) are reported. All regressions also adjust for baseline age, race, education, whether individual had diabetes, hyperlipidemia, or hypertension at baseline, whether individual smoked at baseline, and death during follow-up. All costs are in 2016 US Dollars.

© 2022 Khan SS et al. *JAMA Network Open.*

eTable 10. Differences in Average Annual Cost in Older Age by Body Mass Index Category in Midlife Stratified by Sex, N=23,342

	**Quantile Regression**			
	**25th %**	**50th %**	**75th %**	**90th %**
**Men, N=13,761**	**Average Annual Costs, $ [95% Confidence Interval]**			
	N=13,761	N=13,761	N=13,761	N=13,761
Underweight, (BMI<18.5 kg/m^2)	0 (-7, 7)	-253 (-991, 485)	-1,141 (-3012, 729)	-4,135 (-19760, 11490)
Normal BMI, (18.5-24.9 kg/m^2)	REFERENT			
Overweight, (BMI 25.0-29.9 kg/m^2)	21 (6, 36)	860 (678, 1043)	1,868 (1328, 2408)	2,592 (1102, 4081)
Obese (BMI 30.0-39.9 kg/m^2)	59 (16, 103)	1,777 (1263, 2290)	4,424 (3350, 5498)	5,863 (3222, 8505)
Morbidly obese (BMI ≥ 40 kg/m^2)	-6 (-164, 153)	56 (-4984, 5095)	5,156 (-2238, 12550)	726 (-120674, 122126)
**Women, N=9,581**	**Average Annual Costs, $ [95% Confidence Interval]**			
	N=9581	N=9581	N=9581	N=9581
Underweight, (BMI<18.5 kg/m^2)	0 (0, 0)	-285 (-428, -141)	-1817 (-2577, -1056)	-4,258 (-6966, -1549)
Normal BMI, (18.5-24.9 kg/m^2)	REFERENT			
Overweight, (BMI 25.0-29.9 kg/m^2)	0 (-1, 1)	837 (433, 1241)	1,795 (1002, 2588)	2,674 (967, 4380)
Obese (BMI 30.0-39.9 kg/m^2)	41 (23, 58)	2,737 (1896, 3579)	6,422 (4344, 8500)	12,646 (5418, 19874)
Morbidly obese (BMI ≥ 40 kg/m^2)	0 (-32, 32)	3,581 (-955, 8117)	15,241 (-5852, 36334)	45,578 (-40801, 131956)

Notes: Each column is from a separate regression. Coefficients (cost differences) and 95% confidence intervals (in parentheses) are reported. All regressions also adjust for baseline age, race, education, whether individual had diabetes, hyperlipidemia, or hypertension at baseline, whether individual smoked at baseline, and death during follow-up. All costs are in 2016 US Dollars.

© 2022 Khan SS et al. *JAMA Network Open.*

eTable 11. Differences in Cumulative Cost and Average Annual Cost in Older Age by Body Mass Index Category in Midlife in All Participants With Available Medicare Data, N=29,621

	Quantile Regression			
**N=29,621**	**25th %**	**50th %**	**75th %**	**90th %**
**Cumulative Costs, 95% Confidence Interval**				
Underweight, (BMI<18.5 kg/m^2)	0 (-92, 92)	-3,659 (-4737, -2580)	-26,663 (-33407, -19920)	-79,498 (-103555, -55440)
Normal BMI[a] (18.5-24.9 kg/m^2)	REFERENT			
Overweight, (BMI 25.0-29.9 kg/m^2)	450 (395,505)	13,813 (11,835, 15,790)	34,719 (28,999, 40,439)	48,520 (37,792, 59,247)
Obese (BMI 30.0-39.9 kg/m^2)	193 (67, 319)	20,347 (15,485, 25,209)	58,542 (45,977, 71,107)	97,603 (76,964, 118,241)
Morbidly obese (BMI ≥ 40 kg/m^2)	0 (-786, 786)	21,644 (-11,267, 54,556)	83,606 (16,734, 150,477)	110,477 (-37,554, 258,508)
**Average Annual Costs, 95% Confidence Interval**				
Underweight, (BMI<18.5 kg/m^2)	0 (-1, 1)	-164 (-293, -36)	-1,549 (-2,411, -686)	-3,786 (-6013, -1558)
Normal BMI[b] (18.5-24.9 kg/m^2)	REFERENT			
Overweight, (BMI 25.0-29.9 kg/m^2)	56 (53, 59)	922 (743, 1,101)	1,612 (1,264, 1,961)	2,153 (1,213, 3,092)
Obese (BMI 30.0-39.9 kg/m^2)	40 (25, 54)	1,850 (1,460, 2,239)	4,284 (3,541, 5,027)	7,207 (5,366, 9,048)
Morbidly obese (BMI ≥ 40 kg/m^2)	0 (-47, 47)	3,374 (136, 6,612)	8,401 (2,169, 14,633)	30,270 (-9,528, 70,068)

Notes: Each column is from a separate regression. Coefficients (cost differences) and 95% confidence intervals (in parentheses) are reported. All regressions also adjust for baseline age, race, sex, education, whether individual had diabetes, hyperlipidemia, or hypertension at baseline, whether individual smoked at baseline, and death. Costs are in 2016 US Dollars. BMI reflects body mass index

a) For the referent group, cumulative costs for normal BMI at the 25th, 50th, 75th, and 90th percentiles were $148,531, $325,254, $610,651, and $950,934, respectively
b) For the referent group, average annual costs for normal BMI at the 25th, 50th, 75th, and 90th percentiles were $11,318, $23,529, $42,577, and $76,796

© 2022 Khan SS et al. *JAMA Network Open.*

eTable 12. Differences in Cumulative Cost and Average Annual Cost in Older Age by Body Mass Index Category in Midlife Excluding Individuals With Zero Costs*, N=23,353

	**Quantile Regression**			
**N=23,353**	**25th %**	**50th %**	**75th %**	**90th %**
**Cumulative Costs, 95% Confidence Interval**				
Cost of Reference group (Normal BMI)	$78,842	$255,659	$585,440	$976,362
Underweight, (BMI<18.5 kg/m^2)	-1,430 (-2656, -205)	-10498 (-15439, -5556)	-44801 (-63597, -26004)	-78,013 (-104193, -51832)
Normal BMI, (18.5-24.9 kg/m^2)	REFERENT			
Overweight, (BMI 25.0-29.9 kg/m^2)	4,837 (3502, 6,172)	17,481 (13377, 21585)	29,645 (22149, 37140)	44,756 (32350, 57162)
Obese (BMI 30.0-39.9 kg/m^2)	4,836 (2330, 7342)	28,826 (21554, 36098)	61,185 (46942, 75428)	97,842 (76690, 118995)
Morbidly obese (BMI ≥ 40 kg/m^2)	9852 (-9827, 29532)	60,793 (12682, 108905)	115,023 (52517, 177529)	138,468 (-59239, 336175)
**Average Annual Costs, 95% Confidence Interval**				
Cost of Reference Group (Normal BMI)	$6,730	$19,048	$44,675	$87,095
Underweight, (BMI<18.5 kg/m^2)	-12 (-228, 203)	-336 (-936, 264)	-1,035 (-2157, 87)	-4 (-5144, 5136)
Normal BMI, (18.5-24.9 kg/m^2)	REFERENT			
Overweight, (BMI 25.0-29.9 kg/m^2)	297 (181, 412)	917 (628, 1206)	1,258 (750, 1766)	1,410 (293, 2526)
Obese (BMI 30.0-39.9 kg/m^2)	552 (279, 825)	2,388 (1928, 2847)	4,489 (3618, 5361)	7,946 (6028, 9865)
Morbidly obese (BMI ≥ 40 kg/m^2)	1,208 (-917, 3332)	5,006 (1818, 8194)	11,982 (3443, 20521)	30,343 (-44444, 105131)

Notes: Sample limited to individuals with non-zero costs in any year. Each column is from a separate regression. Coefficients (cost differences) and 95% confidence intervals (in parentheses) are reported. All regressions also adjust for baseline age, race, sex, education, whether individual had diabetes, hyperlipidemia, or hypertension at baseline, whether individual smoked at baseline, and death. All costs are in 2016 US Dollars.

© 2022 Khan SS et al. *JAMA Network Open.*
